# Recurrent Miscarriage and Implantation Failure of Unknown Cause Studied by a Panel of Thrombophilia Conditions: Increased Frequency of FXIII Val34Leu Polymorphism

**Published:** 2019

**Authors:** Maria Diaz-Nuñez, Aintzane Rabanal, Antonia Expósito, Marcos Ferrando, Fernando Quintana, Jose Manuel Soria, Roberto Matorras

**Affiliations:** 1- Human Reproduction Unit, Cruces University Hospital, Biocruces, Barakaldo, Spain; 2- IVI Bilbao, Bizkaia, Spain; 3- Unit of Genomics of Complex Diseases, Sant Pau Institute of Biomedical Research (IIB-Sant Pau), Barcelona, Spain

**Keywords:** Factor XII, Implant failure, IVF, Miscarriage, Thrombophilia

## Abstract

**Background:**

The role of acquired thrombophilia has been accepted as an etiology of recurrent miscarriage (RM); however, the contribution of specific inherited thrombophilic genes to this disorder has remained controversial. An increased incidence of RM has been suggested in women with inherited thrombophilia.

**Methods:**

In this prospective study, assisted women with RM or repeated implant failure (RIF) were subjected to Thromboincode analysis, in order to identify 12 genetic variants for Factor V Leiden, Factor V Hong Kong, Factor V Cambridge, FII, FXIII, FXII, and A1 carriers. Patients included in this study were separated in RM cases (n=43), RIF cases (n=36) and RIF+RM (n=76). As a control group, patients undergoing IVF treatment (n=34) were used and a previously described 249 cases population as a representative sample of Spanish population were selected. Level of statistical significance was p<0.05 and groups were compared by Fisher test, except for age that was compared by t-test.

**Results:**

Regarding FXIII, higher values were observed in RM (69.76%), RIF (70%) and in RM+RIF (68.42%) group when compared with our control group (52.94%) and general Spanish population (56.5%), but these differences were statistically significant only in RIF group (p=0.043, p=0.01).

**Conclusion:**

Concerning our findings, both RM and RIF patients had a very similar panel of thrombophilia polymorphisms, suggesting that, in both, thrombophilia might have an important contribution. High frequency of Val34Leu polymorphism in RM/ RIF presumably speaks in favor of a multifactorial RM genesis, wherean altered thrombophilia status plays a role.

## Introduction

Recurrent miscarriage (RM) -defined by ESHRE guideline as ≥2 consecutive pregnancy losses before 20 weeks post menstruation affects approximately 1% of couples trying to conceive (1). Current diagnostic procedures can identify etio-logic factors in approximately 50% of these couples, such as uterine defects, advanced woman age, parental karyotype abnormalities, embryonic aneuploidies, infections and thrombophilia disorders (2, 3). While the role of acquired thrombophilia has been accepted as an etiology of RM, the contribution of specific inherited thrombophilic genes to this disorder has remained controversial (4). An increased incidence of RM has been suggested in women with inherited thrombophilia, including Factor V Leiden deficiency, activated protein C resistance, prothrombin G20210A and protein S deficiency (5–8). Other coagulation abnormalities, such as impaired fibrinolytic activity, factor XII deficiency and reduced activated partial thromboplastin time have also been reported to be associated with RM, but the corresponding epidemiological data are limited (9).

Inherited thrombophilia has the potential to disrupt the natural coagulation system and predispose certain individuals to suffer from a thrombotic event. The mechanism by which thrombophilia causes RM is uncertain but it seems to include thrombosis of the uteroplacental circulation.

On the other hand, repeated implantation failure (RIF)-defined as the transfer of several good quality embryos in IVF cycles, without achieving pregnancy-may share some of the same mechanisms with RM (10, 11). There is no universally accepted definition of RIF despite many publications on this topic, but one of the most used ones is failure of pregnancy after the transfer of 3 good quality embryos (12).

Various investigators have shown that thrombophilia is more common in women with RIF compared with healthy fertile controls (6, 11, 13–15). This association is explained by thrombophilia causing microthrombosis at the implantation site and thereby impairing the initial invasion of maternal vessels by the syncytiotrophoblast, leading to implantation failure (13, 14, 16, 17). However, other investigators have reported no relationship between thrombophilia and recurrent IF (18, 19).

In the last years, a number of thrombophilia conditions related with thrombosis risk in the adult have been described (20). Recently, a commercially available kit (Thromboincode, Laboratorios Ferrer, Barcelona) has been introduced, allowing to analyze 12 low frequency, high impact genetic coagulation disorders in the same saliva sample (20). The analysis studies the alleles of 12 variants located in seven genes (PT, FVL, FXII, FXIII, ABO, SERPIN A10 and SERPIN C1). This test accurately determined the thrombosis risk in adult patients (20).

It has been suggested that only 15% of women with thrombophilic risk factors contributing to the RM would be identified if only mutations in factor V von Leiden, factor II (Prothrombin) and MTHFR were investigated (21). On the other hand, it has been shown that while none of the specific thrombophilic gene mutations appeared to be a risk factor for RM, when taken together, the total number of mutations was a significant risk (5). Thus, our objective was to analyze the results of the aforementioned 12 polymorphism panel in our RM/RIF patients where all the conventional studies were normal.

## Methods

This is a prospective study performed in two centers (The Human Reproduction Unit of the Cruces University Hospital and the IVI Clinic Bilbao, Spain) in which women were enrolled between June 2014 and June 2015. During this twelve month period, all the couples assisted because of RM or RIF whose standard study was normal, were subjected to Thromboincode analysis.

Inclusion criteria were: i) at least 2 previous clinical miscarriages (In RM cases) or at least 2 transfers with at least 1 high-quality embryo in each without achieving pregnancy (In RIF cases) ii) woman age <40 years (<45 in cases of oocyte donation) iii) body mass index <35, and iv) conventional RM/RIF study showing no abnormalities. Conventional RM/RIF study included all of the following woman and man karyotypes, vaginal ultrasonography, hysterosalpingography, hysteroscopy, sperm DNA fragmentation, homocysteinemia, thyroid hormones, fasting glucose, anti-phonspholipid study, and “habitual thrombophilia study”. Patients with polycystic ovarian syndrome or autoimmune disorders were excluded, as well as those with a history of preeclampsia, cardiac disease or thrombosis. Habitual thrombophilia study consisted of Factor V Leiden deficiency, activated protein C resistance, prothrombin G20210A, and protein S deficiency methylenetetrahydrofolate reductase (MTHFR) mutations. Ethical approval was obtained from the Clinical Research Ethical Committee of the Basque Health System (Osakidetza, CEIC reference no E16/48).

Informed consent was obtained from all patients included in this study were separated in RM cases (n=43), RIF cases (n=36) and RIF+RM (n=76). As a control group, 34 IVF women undergoing their first IVF cycle, aged <40 years (<45 in cases of oocyte donation) with body mass index <35 and without venous thrombosis history were included. Due to the small number of patients in the control group, our results were also compared with a previously described 249 case population corresponding to a representative sample of the Spanish population (20). Mean age was 46±14.9 years, and 56% were females. All of them were healthy and with no venous thrombosis history.

Thromboincode rationale was based in a previous systematic review and meta-analysis performed to select genetic variants that contribute to venous thrombotic risk (20). Based on this information, a panel with the variants rs6025 (FV, Factor V Leiden), rs118203906 (FV, Factor V Hong Kong), rs118203905 (FV, Factor V Cambridge), rs1799963 (FII, G20210A), rs5985 (FXIII, V34L), rs121909548 (SERPINC1, 384 Ala>Ser), rs2232698 (SERPINA10, 67 ARG>Stop), and rs1801020 (FXII, 46 C>T) and the A1 carriers rs8176719, rs78539 89, rs8176743, and rs8176750 was defined. The ThromboinCode kit (FererinCode) was used to identify the variants included in this panel. It is important to note that all of these genetic variants have functional effects on the coagulation cascade. All except the A1 carriers are gain or loss-of function variants. ThromboinCode® (TiC) enables diagnosis of hereditary thrombophilia (By analyzing genetic variants affecting different points of the blood coagulation cascade function, promoting the development of VTE) and is also a clinical genetic function for assessing the risk of VTE.

It has to be noticed that all of our patients have been previously tested to factor V Leiden and prothrombin mutation, and to be included in the study they had to be normal.

Since a number of polymorphisms were investigated, each of them with a very different prevalence in the control group, and with, if any, a different increase in the study patients, no sample size calculation was done. In deed some of the polymorphisms (SERPIN A10 and C1) have not been previously tested in RM/RIF.

For the statistical analysis, the results obtained in the RM and RIF were compared separately with the control group. Later, RM/RIF was compared with the control cases and RM cases was compared with RIF cases. Finally, RM, RIF and RIF /RM were compared with general Spanish population described by Soria (20). Comparisons were made with Fisher exact test, following the standard criteria of applicability and age was compared using t-test. The odds ratio (OR), its standard error and 95% confidence interval were calculated according to Altman (1991) (22), while zeros cause problems with computation of the odds ratio or its standard error, 0.5 is added to all cells (23, 24). Significance was established at p<0.05.

Calculation of costs was made based on following costs of the market analysis in the private center which include Factor V Leiden study (160 Euros), prothrombin study (160 euros), Thromboincode (277 euros).

### Ethical consideration:

Ethics approval and consent to participate: Institutional Board approval (CEIC reference no. E16/48) and informed consent were obtained.

## Results

All groups in our study showed similar age but it was significantly lower than the age in the population described by Soria (Table 1). Also the prevalence of smokers was higher in general Spanish population.

**Table 1.** Thromboincode results and epidemiological data

	**RIF+RM n=76**	**RIF n=36**	**RM n=43**	**Infertile Control population n=34**	**General Spanish population n=249**	**p**	**OR**	**CI**
**Age**	37,07±3,71	37,7±3,73	36,79±3,7	36,75±3,15	49.0±14.9	0.66^a^ 0.25^b^ 0.42^c^ *0.001^a′^^b′^^c′^	N/A	N/A
**Smoking**	9 (11,84%)	3 (7,5%)	6 (13,95%)	1 (2,94%)	101 (40.7%)	0.17^a^ 0.61^b^ 0.12^c^ *0.0001^a′^^b′^ *0.0006^c′^	N/A	N/A
**ABO-A1 carriers**	22 (28,94%)	14 (35%)	9 (20,93%)	14 (41.17%)	87 (35.7%)	0.27^a^ 0.4^a′^ 1^b^ 0.71^b′^ 0.07^c^ 0.08^c′^	N/A	N/A
**FXII (T) rs1801020**	0	0	0	2 (5,05%)	5 (2.02%)	0.09^a^ 0.59^a′^ 0.23^b^ 1^b′^ 0.19^c^ 1^c′^	N/A	N/A
**SERPIN A10 (T) rs2232698**	1 (1,31%)	1 (2,5%)	0	0	4 (1.61%)	1^a, a′^ 1^b^ 0.49^b′^ 1^c, c′^	N/A	N/A
**SERPIN C1 (T) rs121909548**	1 (1,31%)	1 (2,5%)	0	0	1 (0.40%)	1^a,^ 0.41^a′^ 1^b^ 0.23^b′^ 1^c, c′^	N/A	N/A
**FXIII (G) rs5985**	52 (68,42%)	28 (70%)	30 (69,76%)	18 (52,94%)	139 (56.5%)	0.13^a^ 0.06^a^^b^ * 0.043^b^ 0.01 0.15^c^ 0.09^c^	1.92^a^ 2.76^b^ 2.05^c^	0.84–4.41^a^ 1.21–6.31^b^ 0.80–5.23^c^

a: RIF+RM *vs*. control;

a′: RIF+RM *vs*. general Spanish population;

b: RIF *vs*. control;

b′: RIF *vs*. general Spanish population;

c: RM *vs*. control;

c′: RM *vs*. general Spanish population)

No significant differences were observed between groups when studying A1 carriers, FXII, SERPIN C1 or SERPIN E10 polymorphisms. In fact, these polymorphisms showed a very similar pattern in all groups.

Regarding FXIII, a trend to higher values was observed in RM (69,76%) and RIF/RM group (68,42%) when compared with our control group (52.94%), but significant differences were only found in the RIF cases (70%) (p=0.043) (OR= 2.76, CI=1.21–6.31). Also when comparing our control group with the general Spanish population, it was found that values in both control groups are similar and the differences between RIF and population control reached significance again (p=0.01). Nevertheless, it has to be highlighted that frequencies in RM, RIF and RM/RIF group were very similar, so statistical differences might be found only by increasing the number of patients.

On the other hand, as expected, no FV or FII cases were observed in the study groups (Data not shown), since all the patients to be included in our study had to be previously tested (And negative) for FV and FII.

When comparing RM with RIF group, a very similar pattern in all the polymorphisms studied was observed.

## Discussion

Both RM and RIF may be caused by a number of conditions, but standard diagnostic work-ups fail to find a cause in a number of them. In recent years, thrombophilia study has received an increased attention in RM/RIF study.

Although results are controversial, usual standard inherited thrombophilia RM/RIF study includes Factor V Leiden deficiency, activated protein C resistance, prothrombin G20210A and protein S deficiency and, sometimes methylenetetrahydrofolate reductase mutations (5–7, 11). Other coagulation abnormalities, including impaired fibrinolytic activity, factor XII deficiency and reduced activated partial thromboplastin time have also been reported to be associated with RM, but the corresponding epidemiological data are limited (9).

Notwithstanding, even applying conventional thrombophilia studies, RM/RIF study is frustrating for both the couple and the medical team, since in close to 50%, no cause is diagnosed. Regarding costs, Thromboincode costs were 266 euros, which is less expensive than performing separately the analysis of factor V Leiden and prothrombin mutation (320 euros=160+160), which are included in Thromboincode analysis.

This is the reason why we subjected our couples to an extended thrombophilia detection which accurately predicted thrombosis events in the adult. Our test studied 5 single nucleotide polymorphisms (SNP) with the highest individual odds ratio for venous thrombosis (25) (Factor V Leiden, PT G20210A, A1 blood group, 1 SNP in the Fibrinogen -gene, 1 in the Factor XI gene) as well as some rare genetic variants linked to the thrombosis risk, with stronger effects in the carriers, such as the SERPIN C1 gene (26), the R67X in SERPIN A10 gene (27), or Ser219Gly in the PROCR gene (28). There is a strong belief that RSA patients with unknown etiology have a multifactorial condition and that genetic and environmental elements play a key role (9).

It should be highlighted that two of the more prevalent thrombophilia types (Factor V deficiency and prothrombin mutation), which are included in the Thromboincode were previously systematically analyzed in our patients, constituting an exclusion criteria for the present study.

There might be some concern regarding the composition of the Spanish population group, where about 56% of the cases were males, and where the mean age was 12 years higher than the one in the study group. Also, there are fewer smokers in the study groups, probably because patients included in our study were young women trying to achieve pregnancy. Regarding gender, it has to be highlighted that in the healthy population, there are no differences in the prevalence of thrombophilia conditions, as in the majority of genetic polymorphisms. Concerning the higher age in the control group, in our opinion, it was the strength of our study design. Some patients that perhaps could be part of the healthy control group at a young age, could be carrier of a thrombophilia type that some years later could have experienced some vascular injuries or coagulation and then not fulfilling the control criteria at an older age. In agreement with this, some authors have constituted their control group only with post-menopausal women (29).

Concerning our findings, it has to be highlighted that both RM and RIF patients had a very similar panel of thrombophilia polymorphisms, suggesting that, in both, thrombophilia plays a similar role, at least for the polymorphisms studied before.

The most relevant finding of our study was the increased frequency of FXIII Val34Leu polymorphism in the RM and RIF cases when compared with the control group. The main hemostatic function of FXIII is the mechanical stabilization of fibrin clot and the protection of newly formed fibrin from the prompt elimination by fibrinolysis (30). Its importance is underlined by the severe bleeding diathesis of patients with severe FXIII deficiency (31, 32). However, FXIII also exerts antithrombotic effect by the inhibition of platelet adhesion to fibrin polymers (30). The most common FXIII polymorphisms is the point mutation of G → T in the 2nd exon of the FXIII A-subunit gene, that leads to conversion of Val to Leu within codon 34 (FXIII Val34Leu). This polymorphism produces an acceleration of the activation of FXIII (30, 33, 34) and impacts the clot stability and cross-linking activity (35, 36). Besides, the polymorphism also influences the structure of fibrin in a fibrinogen concentration dependent manner (37). At high fibrinogen concentration, plasma samples from individuals homozygous for the Leu allele form clot with thicker fibers, increased permeability, looser structure and increased susceptibility to fibrinolysis than plasma clots from wild type individuals. Furthermore, FXIII is in wound healing and angiogenesis (30).

Although there is considerable variation among different series, in a number of meta-analyses it has been shown that the polymorphism FXIII-A Val34Leu has a protective effect on the incidence of coronary disease and myocardial infarction (38).

The relationship of Val34Leu with RM is controversial (39–41). However, in a very recent meta-analysis, it has been shown that FXIII-A Val34Leu is associated with an increased risk of RM (9). Part of the discrepancies in studies analyzing the relationship between RM and Val/Leu FXIII polymorphism could be related with the remarkable differences in the frequency of the polymorphism Val34/Leu in the general population. It has been reported that the Leu34 allele is present in approximately 25% of Europeans, much less frequent in Africans and it is missed in Asians (International HapMap project, www.hapmap.org) (30). Surprisingly, almost 70% of our study patients were carriers of the Val34/Leu polymorphism, although this was statistically significant only for the RIF group (OR=2.76, CI=1.21–6.31), since in our control group there was a high frequency of Val34/Leu carriers. RM and RM+RIF groups, even if they do not reach statistical significance, probably due to the modest number of control cases, OR values should be outlined (OR=2.05, CI=0.80–5.23 and OR=1.92, CI=0.84–4.41).

In our opinion, the reported findings of the high frequency of Val34/Leu polymorphism in RM and RIF presumably speak in favor of a multifactorial RM genesis, where an altered thrombophilia status plays a role. The analysis of multiple genes gives help in the understanding of complex situation of RM+RIF.

The importance of the gene–gene, SNP–SNP, and gene environment interactions should be taken into account (9), as well as the possibility that poor pregnancy outcomes may also be associated with fetal thrombophilia by inheritance of maternal and paternal genes (21).

## Conclusion

Concerning our findings, both RM and RIF patients had a very similar panel of thrombophilia polymorphisms, suggesting that, in both, thrombophilia might have an important contribution. High frequency of Val34Leu polymorphism in RM/RIF presumably speaks in favor of a multi-factorial RM genesis, where an altered thrombophilia status plays a role.
